# GLP toxicology study of a fully-human T cell redirecting CD3:EGFRvIII binding immunotherapeutic bispecific antibody

**DOI:** 10.1371/journal.pone.0236374

**Published:** 2020-07-31

**Authors:** Patrick C. Gedeon, Michael A. Streicker, Teilo H. Schaller, Gary E. Archer, Micheal P. Jokinen, John H. Sampson

**Affiliations:** 1 Duke Brain Tumor Immunotherapy Program, Department of Neurosurgery, Duke University Medical Center, Durham, NC, United States of America; 2 Department of Biomedical Engineering, Duke University, Durham, NC, United States of America; 3 The Preston Robert Tisch Brain Tumor Center, Department of Neurosurgery, Duke University Medical Center, Durham, NC, United States of America; 4 Integrated Laboratory Systems, Inc., Research Triangle Park, NC, United States of America; 5 Department of Pathology, Duke University Medical Center, Durham, NC, United States of America; Sechenov First Medical University, RUSSIAN FEDERATION

## Abstract

We recently reported the development of a fully-human, CD3-binding bispecific antibody for immunotherapy of malignant glioma. To translate this therapeutic (hEGFRvIII-CD3- bi-scFv) to clinical trials and to help further the translation of other similar CD3-binding therapeutics, some of which are associated with neurologic toxicities, we performed a good laboratory practice (GLP) toxicity study to assess for potential behavioral, chemical, hematologic, and pathologic toxicities including evaluation for experimental autoimmune encephalomyelitis (EAE). To perform this study, male and female C57/BL6 mice heterozygous for the human CD3 transgene (20/sex) were allocated to one of four designated groups. All animals were administered one dose level of hEGFRvIII-CD3 bi-scFv or vehicle control. Test groups were monitored for feed consumption, changes in body weight, and behavioral disturbances including signs of EAE. Urinalysis, hematologic, and clinical chemistry analysis were also performed. Vehicle and test chemical-treated groups were humanely euthanized 48 hours or 14 days following dose administration. Complete gross necropsy of all tissues was performed, and selected tissues plus all observed gross lesions were collected and evaluated for microscopic changes. This included hematoxylin-eosin histopathological evaluation and Fe-ECR staining for myelin sheath enumeration. There were no abnormal clinical observations or signs of EAE noted during the study. There were no statistical changes in food consumption, body weight gain, or final body weight among groups exposed to hEGFRvIII-CD3 bi-scFv compared to the control groups for the 2- and 14-day timepoints. There were statistical differences in some clinical chemistry, hematologic and urinalysis endpoints, primarily in the females at the 14-day timepoint (hematocrit, calcium, phosphorous, and total protein). No pathological findings related to hEGFRvIII-CD3 bi-scFv administration were observed. A number of gross and microscopic observations were noted but all were considered to be incidental background findings. The results of this study allow for further translation of this and other important CD3 modulating bispecific antibodies.

## Introduction

We have recently reported the pre-clinical development of a fully-human EGFRvIII:CD3 binding bispecific antibody (hEGFRvIII-CD3 bi-scFv) that effectively redirects human T cells to lyse patient derived malignant glioma expressing the tumor specific mutation of the epidermal growth factor receptor (EGFRvIII) [1]. Such bispecific antibody based therapy promises to overcome many critical barriers that have traditionally limited translation of immunotherapy to the clinic, as evidence by FDA approvals of blinatumomab [2–4], for example, a CD3:CD19 binding bispecific antibody, and many other similar CD3 binding bispecific antibodies that are currently under development [5]. These CD3 modulating therapeutics, however, are associated with adverse neurologic events [3, 6–8]. To further explore this important phenomenon, and to advance hEGFRvIII-CD3 bi-scFv as a safe and effective therapeutic for patients with malignant glioma, we report here the results of a good laboratory practice (GLP) toxicology study of hEGFRvIII-CD3 bi-scFv.

We have previously demonstrated that intravenously administered hEGFRvIII-CD3 bi-scFv accumulates to therapeutic levels in highly-invasive, syngeneic, tumors within the brain, leading to durable cures among cohorts of mice with well-established tumors [1]. To most expeditiously assess and validate this straightforward and clinically feasible mechanism of drug delivery among patients with malignant glioma, we have conducted an extended single-dose toxicity study as recommended by the US Food and Drug Administration’s (FDA) *Guidance for Industry*, *Investigators*, *and Reviewers for Exploratory IND* Studies. While conducted rigorously and under strict GLP practices, restriction of the toxicity study to a single dose, as recommended by the US FDA in cases where the anticipated clinical trial involves the goal of collecting pharmacokinetic information or performing imaging studies [9], allowed for a reduction of the time and resources expended prior to clinical assessment while maintaining the stringent requirements needed for human subject protection and initiation of clinical study.

We have furthermore implemented a unique human CD3 transgenic mouse model. Given that the CD3 binding portion of hEGFRvIII-CD3 bi-scFv does not bind to CD3 in other species including non-human primates [1, 10], we have utilized a human CD3 transgenic mouse model that is pharmacologically responsive to hEGFRvIII-CD3 bi-scFv. Signaling via human CD3 receptors on the surface of these transgenic T cells induces a signaling cascade and functional outcomes similar to native murine CD3 engagement [1, 11, 12]. Use of this model for toxicity evaluation allows for a pre-clinical toxicology study in a pharmacologically responsive animal model when it would otherwise not be possible.

## Materials and methods

The purpose of the study was to determine the toxicity of hEGFRvIII-CD3 bi-scFv administered by intravenous injection to study animals pharmacologically responsive to the hEGFRvIII-CD3 bi-scFv antibody. The study was conducted in accordance with U.S. Food and Drug Administration’s Good Laboratory Practice Regulations (21 CFR Part 58). Analysis of potential toxicity included: clinical observation with formal assessment for EAE; analysis of feed consumption and body weight; urinalysis; hematologic assessment; clinical chemistry; and macroscopic and microscopic pathologic assessment including standard histopathologic assessment and myelin sheath enumeration.

### Study drug

The product is a recombinant bispecific antibody fragment which binds to human CD3 on T cells and a tumor specific mutation, EGFRvIII. When engaged with both T cells and tumor cell targets, the recombinant antibody induces T cell proliferation, secretion of pro-inflammatory cytokines, T cell activation, and tumor cell lysis [1]. hEGFRvIII-CD3 bi-scFv was prepared in formulation buffer (1.0588 mM KH_2_PO_4_; 155 mM NaCl; 2.966 mM Na_2_HPO_4_-7H_2_O; and 0.01% polysorbate 80) at a maximal feasible concentration of 0.75 mg/mL. Prior to release, the study drug was subject to visual inspection (clear, colorless liquid), pH assessment (7.4), and analysis of protein concentration via Bradford assay. Additionally, prior to release the study drug was validated for target binding via both surface plasmon resonance (SPR) and fluorescence activated cell sorting (FACS) and assessed for purity via SDS-PAGE (>95% purity). Dose formulations of hEGFRvIII-CD3 bi-scFv and vehicle (formulation buffer alone) were prepared and stored at 2–6°C and protected from light.

### Study animals

All study animals were bread and genotyped at Duke University (Durham, NC) according to protocols (A283-15-11) approved by the Duke University Institutional Animal Care and Use Committee (IACUC). Prior to study initiation animals were transferred to Integrated Laboratory Systems, Inc. (Research Triangle Park, NC) where the study was conducted according to protocols (2016–24) specifically approved by the IACUC at Integrated Laboratory Systems, Inc. who is licensed by the U.S. Department of Agriculture (USDA) and accredited by the Association for Assessment and Accreditation of Laboratory Animal Care (AAALAC). All study procedures were conducted in compliance with the Animal Welfare Act Regulations, 9 CFR 1–4. Animals were handled and treated according to the Guide for the Care and Use of Laboratory Animals issued by the Institute for Laboratory Animal Research at the National Research Council of the National Academies [13].

Human CD3 transgenic mice heterozygous for the human CD3 transgene were used for study (tgε600, Jackson Laboratory, strain number 20456 crossed with wild-type C57/BL6). This specific mouse model is useful for the testing of this compound given that the CD3-binding portion of hEGFRvIII-CD3 bi-scFv binds only to human CD3 and does not cross react with CD3 in other species including non-human primates.

At the start of the study, mice were 6–7 weeks of age with males weighing from 19.1 to 24.2 grams and females weighing from 15.1 to 19.0 grams. Each animal was uniquely identified by ear tag prior to the start of the study. Until the animals were ear tagged, they were identified by the temporary numbers located on the animals’ enclosures.

### Animal husbandry

Animals were acclimatized for at least five days prior to study initiation. Mice were housed individually in polycarbonate cages with micro-isolator tops. Each cage measured 17 cm wide by 28 cm long (476 cm^2^ area) and 13 cm high. Absorbent heat-treated hardwood bedding (Northeastern Products Corp., Warrensburg, NY) was used with cage changes once per week. Certified Purina Pico Chow No. 5002 Meal (Ralston Purina Co., St. Louis, MO) was provided *ad libitum*. The manufacturer’s composition formula was included in the raw data and reviewed prior to use. Reverse osmosis (RO) treated tap water (City of Durham, NC) was provided *ad libitum* in polycarbonate water bottles with stainless steel sipper tubes. The results of the current annual comprehensive chemical analyses of RO water from National Testing Laboratories, Inc. (Cleveland, OH) were reviewed prior to initiation of the study and was included in the raw data. Water bottles were changed once per week. The ambient temperature was kept between 20.9–23.9°C. A 12/12 hour light/dark cycle was used. Nestlets (Ancare, Bellmont, NY) enrichment was provided with the manufacture’s analytical results of the nestlets included in the raw data and reviewed before study initiation.

### Allocation and groups

Twenty male and twenty female C57/BL6 mice heterozygous for the human CD3 transgene were allocated to one of four designated dose groups (**Table 1**). The animals were assigned to a dose group using a procedure that stratifies animals across groups by body weight such that mean body weight of each group was not statistically different from any other group using analysis of variance (ANOVA) (Statistical Analysis System version 9.2, SAS Institute, Cary, NC). The animals were administered one dose level of hEGFRvIII-CD3 bi-scFv or the vehicle control (formulation buffer). Forty-eight hours following dose administration, animals in groups 1 and 3 were humanely euthanized. Fourteen days after dose administration, animals in groups 2 and 4 were humanely euthanized. Animals were monitored clinically, urine was collected for urinalysis, and blood was collected for hematology and clinical chemistry. At study endpoints animals were humanly euthanized and tissues were collected for histopathological evaluation.

**Table 1:** Group designation, animal identification, and dose levels.

Group Number	N (M/F)	Animal Identification (M/F)		Test Article	Test Article Dose Level	Day of Termination
1	5/5	01–05	06–10	Vehicle Control	0 mg/kg	2
2	5/5	11–15*	16–20	Vehicle Control	0 mg/kg	14
3	5/5	21–25	26–30*	hEGFRvIII-CD3 bi-scFv	7.5 mg/kg	2
4	5/5	31–35	36–40	hEGFRvIII-CD3 bi-scFv	7.5 mg/kg	14

*At dosing, animal 41 replaced animal 11 and animal 42 replaced animal 29.

### Test article administration, route, dosage

Dose formulations were administered once on day 0 via intravenous injection at a dose volume of 10.0 mL/kg. The dosing sequence was stratified across dose groups; one animal per sex from each group and then repeated until all animals were dosed. The dose level selected was the maximal feasible dose given the maximum feasible concentration of the test article and the maximum acceptable intravenous volume that could be administered. Dose formulations were disposed of as non-hazardous material. Test and control articles are maintained for a period of five years following finalization of the study report.

### In-life animal observations, EAE assessment, feed consumption, weights and urinalysis

Animals were observed for allocation and then daily beginning on study day 0. Observations focused on ambulation, including the ability to ambulate to food and water and also to move forward 2 steps when touched. EAE observations were performed twice daily (once in the morning and once in the afternoon) using the following scale: no signs (0), limp tail (1), hind-limb weakness (2), paraplegia (3), and quadriplegia or moribund (4). Groups 3 and 4 were evaluated for statistically significant EAE scores compared to the control groups (1 and 2) treated with formulation buffer. Body weights were collected prior to study initiation for allocation then daily beginning on day 0. Feed consumption was measured weekly on the same days body weights were collected beginning at dose administration. For urinalysis, each animal was fasted overnight and an 18-hour urine sample (kept cold during collection) was collected overnight prior to necropsy. Volume and appearance were recorded at the time of collection. Urine was stored at or below 10°C. Urine was sent to Antech GLP in Morrisville, NC 27560 for analysis.

### Termination, hematology, and clinical chemistry

Approximately 48 hours (± 30 minutes) after dose administration, animals from groups 1 and 3 were euthanized by CO_2_ asphyxiation and death confirmed by exsanguination. Approximately 14 days (± 30 minutes) after dose administration, animals from groups 2 and 4 were euthanized by CO_2_ asphyxiation and death confirmed by exsanguination.

Blood was collected via cardiac puncture and discharged into an EDTA tube (inverted and stored on wet ice) and serum separator tube. The serum was allowed to clot at room temperature for at least a half hour; blood was centrifuged at 1000 g for 15 minutes and serum poured off into labeled 1.2 mL cryogenic vials and frozen at or below -70°C.

Whole blood and serum were sent to Antech GLP in Morrisville, NC 27560 for analysis. For hematological analysis a complete blood count, including platelet and differential count was performed. For clinical chemistry, serum samples were evaluated for the following endpoints: total protein, albumin, alanine aminotransferase, aspartate aminotransferase, alkaline phosphatase, gamma glutamyl transferase, total bilirubin, sorbitol dehydrogenase, sodium, potassium, chloride, calcium, phosphorous, creatinine, and blood urea nitrogen. For urine, specific gravity, pH, glucose, protein, occult blood, ketones, bilirubin, and urobilinogen were recorded/evaluated and the sediment examined by microscopy.

### Pathology

Complete postmortem examinations were performed on all animals [14]. For each animal, a section of the left lobe of the liver, heart, lung (insufflated), brain, spleen, thymus, inguinal lymph nodes, mesenteric lymph nodes, both kidneys, and all gross lesions observed at necropsy were fixed in 10% neutral buffered formalin NBF for 18–24 hours and then transferred to 70% histology grade alcohol prior to paraffin embedding. Microscopic examination of routinely prepared hematoxylin-eosin stained paraffin sections was performed on all tissues collected at necropsy from all animals in all groups. In addition, three sections of brain per animal were stained with Fe-ECR stain for myelin sheath evaluation. The number of discrete demyelinating plaques were compared between groups. Stained histologic sections were examined by light microscopy and observations were entered into Provantis by the study pathologist. Histologic sections were of adequate size and quality for detailed evaluation, and the number of tissues examined from each treatment group was considered sufficient to allow detection of test article-related histologic alterations. Histopathologic lesions were classified using standard published terminology to the extent possible. Lesions were graded as to severity. The Provantis histopathology tables contain all the recorded data.

### Statistical analysis

All data were analyzed using Statistical Analysis System version 9.2 (SAS Institute, Cary, NC). To perform the analysis male (n = 20) and female (n = 20) mice were assigned to one of four designated dose groups by stratifying animals across groups by body weight such that the mean body weight of each group was not statistically different from any other group by ANOVA. Individual animal data and group means and standard deviations were calculated and reported for each study outcome variable. In cases where insufficient sample was obtained, an annotation of quantity not sufficient is included in the individual animal data. In such cases, the study animal was removed from mean and standard deviation analysis for the specific study variable for which data could not be obtained. Final body weight and body weight gain measurements, food consumption, EAE observations, clinical chemistry data, urinalysis data, and pathology (number of discrete demyelinating plaques) were analyzed. Homogeneity of variance was analyzed using Levene’s test and outliers determined using residual plots. Homogenous data were analyzed using a one-way analysis of variance (ANOVA) and test-article administered groups compared to the appropriate control group using Dunnett’s test. Data that were not homogenous were transformed and re-assessed. In the event data could not be transformed to be homogenous the data were analyzed using the appropriate non-parametric Dunn’s test.

## Results

### In life animal behavioral and EAE observations

All animals survived to the scheduled termination. During the course of the study there were no abnormal behavioral observations noted with the exception that animal 04 was hunched on Day 2 (male, 0.0 mg/kg, vehicle control group). There were also no signs of EAE noted during the study, with all animals scoring no signs (score of 0) at each of the assessment points throughout the study.

### Body weights

Group mean initial and final body weights, and body weight gain for animals euthanized 2- and 14-days post dose are presented in **Tables 2** (males) and **3** (females). There were no statistical changes in final body weight or body weight gain of groups administered hEGFRvIII-CD3 bi-scFv compared to the concurrent control groups for the 2- and 14-day timepoints.

**Table 2:** Male dose group body weight change.

Group Number	hEGFRvIII-CD3 bi-scFv Dose Level	Day of Termination	Initial Group Mean Body Weight (g) ± SD	Final Group Mean Body Weight (g) ± SD	Mean Body Weight Gain (g) ± SD
1	0 mg/kg	2	21.5 ± 1.1	22.0 ± 0.7	0.6 ± 1.3
2	0 mg/kg	14	20.9 ± 2.1	22.6 ± 1.4	1.7 ± 1.3
3	7.5 mg/kg	2	21.3 ± 1.4	22.1 ± 1.0	0.8 ± 0.6
4	7.5 mg/kg	14	20.8 ± 1.3	22.1 ± 1.2	1.4 ± 1.5

* SD = standard deviation.

**Table 3:** Female dose group body weight change.

Group Number	hEGFRvIII-CD3 bi-scFv Dose Level	Day of Termination	Initial Group Mean Body Weight (g) ± SD	Final Group Mean Body Weight (g) ± SD	Mean Body Weight Gain (g) ± SD#
1	0 mg/kg	2	17.1 ± 1.1	17.9 ± 1.1	0.7 ± 0.8
2	0 mg/kg	14	16.9 ± 1.1	18.7 ± 0.8	1.8 ± 0.9
3	7.5 mg/kg	2	17.0 ± 0.9	17.9 ± 0.9	0.9 ± 0.6
4	7.5 mg/kg	14	17.0 ± 0.6	18.5 ± 1.1	1.6 ± 0.7

### Food consumption

Group mean food consumption (g / kg body weight / day) for animals euthanized after 2 and 14 days on study are presented in **Tables 4** (males) and **5** (females). There were no statistical differences in measured food consumption between groups administered hEGFRvIII-CD3 bi-scFv compared to the concurrent control groups for the 2- and 14-day timepoints.

**Table 4:** Male dose group mean food consumption.

Group Number	hEGFRvIII-CD3 bi-scFv Dose Level	Day of Termination	Mean Food Consumption (g/kg body weight/day) ± SD
1	0 mg/kg	2	233.9 ± 65.4
2	0 mg/kg	14	154.9 ± 19.1
3	7.5 mg/kg	2	224.5 ± 33.9
4	7.5 mg/kg	14	151.0 ± 11.0

**Table 5:** Female dose group mean food consumption.

Group Number	hEGFRvIII-CD3 bi-scFv Dose Level	Day of Termination	Mean Food Consumption (g/kg body weight/day) ± SD
1	0 mg/kg	2	296.7 ± 28.0
2	0 mg/kg	14	184.7 ± 11.0
3	7.5 mg/kg	2	273.7 ± 54.7
4	7.5 mg/kg	14	170.0 ± 42.1

### Urinalysis

There were no statistical differences in urinalysis endpoints between groups administered hEGFRvIII-CD3 bi-scFv compared to the concurrent control groups for the Day 2 timepoint. There was a statistical difference in protein level (p = 0.0034) between male mice administered hEGFRvIII-CD3 bi-scFv and the concurrent control group at the Day 14 timepoint. Individual animal data are listed in **S1 Table of S1 Data**.

### Hematology and clinical chemistry

There were statistical differences in the levels of alkaline phosphatase (males, p = 0.0312) and chloride (females, non-parametric Dunn’s test) between groups administered hEGFRvIII-CD3 bi-scFv compared to the concurrent control groups for the Day 2 timepoint and in percent lymphocytes (males, p = 0.0124), sorbital dehydrogenase (males, non-parametric Dunn’s test), hematocrit (females, p = 0.0362), calcium (females, p = 0.0053), phosphorous (females, non-parametric Dunn’s test), and total protein (females, p = 0.0220) for the Day 14 timepoint. There were some parameters that could not be tested for the Day 2 timepoint as sufficient blood volume was unable to be obtained from the mice. These are noted in the individual animal data listed in **S1 Table of S1 Data**.

### Necropsy and histopathological observations

#### Survival

All animals survived to the scheduled terminal necropsies on day 2 and day 14.

#### Macroscopic observations

The only gross pathology findings noted during necropsy were black discoloration in several spleens that correlated microscopically with the presence of melanin pigment, a common finding in animals with pigmented hair, as well as focal pale discoloration of one liver which correlated microscopically with glycogen deposition within hepatocytes and was considered an incidental background finding. One day 14 evaluation of females administered hEGFRvIII-CD3 bi-scFv had black discoloration of the spleen grossly but no microscopic correlation was observed.

#### Microscopic observations

All protocol required tissues from animals as well as all gross lesions were examined microscopically. No microscopic findings considered to be related to test article administration were observed. The only finding in the brain was a small squamous cyst in one day 2 evaluation control females. Squamous cysts in the brain are a fairly common developmental malformation in mice and considered to be of no significance [15]. No other findings were observed in either the H&E or Fe-ECR stained brain sections (**Table 6**).

**Table 6:** Enumeration of demyelinating plaques in the brain.

**Day 2 evaluation**			
**Vehicle Control Males**		**hEGFRvIII-CD3 bi-scFv Males**	
Number of animals	No. of total plaques	Number of animals	No. of total plaques
5	0	5	0
**Vehicle Control Females**		**hEGFRvIII-CD3 bi-scFv Females**	
5	0	5	0
**Day 14 evaluation**			
**Vehicle Control Males**		**hEGFRvIII-CD3 bi-scFv Males**	
Number of animals	No. of total plaques	Number of animals	No. of total plaques
5	0	5	0
**Vehicle Control Females**		**hEGFRvIII-CD3 bi-scFv Females**	
5	0	5	0

The most common change observed was atypical hyperplasia of the thymus which was observed in nearly all animals, both control and treated. Microscopically it was characterized by a mild to moderate increase in the number of thymic lymphocytes resulting in thickening of the cortex, as well as large numbers of lymphocytes within the medulla which obscured to variable degrees the normally clear demarcation between the cortex and medulla. Prominent apoptosis, characterized by pyknosis and fragmentation of lymphocyte nuclei, was also present in a few thymuses. The hyperplasia was termed atypical due to the diffuse proliferation of lymphocytes which obscured the corticomedullary junction [16]. Normally, thymic hyperplasia affects either the cortex or medulla without affecting the corticomedullary junction. The overall appearance was consistent with thymuses which were highly active with a high level of lymphocyte proliferation, and this was considered to be related to the altered genotype of the animals as it was found across age-matched males and females treated with either vehicle control or hEGFRvIII-CD3 bi-scFv. Indeed, the human CD3 epsilon transgene is known to disrupt T cell development in the murine thymus via signal-transduction molecules recruited by the cytoplasmic tail of the human CD3 epsilon protein [11,12]. A number of animals had thymic cysts and one animal had a piece of ectopic thyroid attached to the capsule. These are common background findings and were considered to be of no significance.

A variety of microscopic changes were observed in various tissues and either occurred with similar incidences in control and high dose animals or sporadically across groups. All were considered to be incidental background changes. The most common was minimal to moderate lymphocytic apoptosis in the mesenteric lymph node which was seen in all groups. Lymphoid apoptosis is a common finding in the lymph nodes of young mice, especially in the mesenteric lymph node, and is considered to be indicative of an active lymph node [16] and of no significance. One mesenteric lymph node also had mild histiocytic infiltrate (histiocytosis). In the spleen, minimal melanin pigmentation, referred to previously as a gross finding in some animals, was seen in eleven animals and minimally increased hematopoiesis was observed in five animals. Minimal focal mixed cell infiltrate and minute focal clusters of mixed inflammatory cells were present in two animals, and mild focal hepatocyte glycogen accumulation, referred to previously as a gross finding, was seen in one animal. Minimal multifocal hepatocyte necrosis was present in one day 2 evaluation female administered hEGFRvIII-CD3 bi-scFv. The cause of the necrosis was undetermined but non-specific hepatocyte necrosis is commonly encountered as an incidental finding in the liver of mice [17]. In the kidney, minimal focal tubular degeneration, minimal proteinaceous cast, and mild pelvic dilation each occurred in a single animal. Tables summarizing intergroup comparisons for all gross and histopathological observations are presented in the **Supporting Information** for day 2 (**S2 Table of S1 Data**) and day 14 (**S3 Table of S1 Data**) animals. In summary, no gross or microscopic findings considered related to test article administration were observed. Atypical hyperplasia of the thymus was present in most animals, both control and treated, and was considered to be related to the altered genotype of the animals. A number of gross and microscopic findings were observed but all were considered to be incidental background findings.

## Discussion

There were no clinical observations noted during the study that would be associated with toxicity. P-value tables for aggregate day 2 and day 14 evaluations are presented in the **S4 and S5 Tables of S1 Data**. No behavioral or pathologic evidence of EAE was observed. There were no statistical changes in food consumption, body weight gain, or final body weight in groups exposed to hEGFRvIII-CD3 bi-scFv compared to the control groups for the 2- and 14-day timepoints. There were statistical differences in some clinical chemistry and urinalysis endpoints, primarily in the females at the 14-day timepoint (hematocrit, calcium, phosphorous, and total protein). These differences are likely due to the group size used and of no biologic significance. This is supported by the fact that similar observations were not noted across sexes at the 14-day timepoint and there is no known biologic rationale for sex specific toxicity due to the study agent. No pathological findings considered to be related to EGFRvIII-CD3 bi-scFv administration were observed, including no demyelinating plaques noted in the brain. A number of gross and microscopic observations were noted, but all were considered to be incidental background findings.

Importantly, there was no neurological toxicity observed in this study, both through behavioral and pathological assessment. Further studies are necessary to determine if hEGFRvIII-CD3 bi-scFv may result in neurological toxicity clinically, as blinatumomab and other CD3 modulating therapeutics have for example, and if so, to further elaborate the mechanism by which this toxicity may occur. The findings presented here, however, will allow for the initiation of such important clinical studies. Additional evaluation of toxicity in the context of prolonged dosing regimens and the presence of target-responsive tumor burden may yield different results. Nonetheless, this extended single-dose GLP toxicity study provides a critical first step in the initiation of an Exploratory IND study that will further validate our anticipated clinical route of administration, pharmacokinetics, and parameters that are most appropriately assessed through clinical study. Of note, as previously mentioned, given that the CD3-binding portion of hEGFRvIII-CD3 bi-scFv does not bind to the CD3 receptor in any non-human species, the use of a human CD3 transgenic mouse model is a unique aspect of this study that has allowed for preclinical assessment of toxicity in a pharmacologically responsive preclinical model where otherwise not possible. Others may wish to also explore the use of this model in the evaluation of CD3 modulating therapeutics to most safely predict toxicity in anticipation of first-in-human clinical studies. Taken together, the results of this GLP, extended single-dose toxicity study further the rationale for continued clinical translation of hEGFRvIII-CD3 bi-scFv as a safe and effective immunotherapeutic for patients with malignant glioma and will allow for expediated advancement to clinical trials while maintaining the stringent requirements needed for patient protection.

## Supporting information

S1 Data (PDF)
