# Supplementary material for: GLP toxicology study of a fully-human T cell redirecting CD3:EGFRvIII binding immunotherapeutic bispecific antibody
Source: PLoS One. 2020 Jul 31;15(7):e0236374. doi: 10.1371/journal.pone.0236374 (PMC7394377; doi:10.1371/journal.pone.0236374)
Supplement: S1 Data — (PDF) [file pone.0236374.s001.pdf]

S1 Table – Individual hematology, clinical chemistry, and urinalysis data

Group: 1 Control Day 2
Males

Animal No.	WBC (1000/μL)	RBC (1000000/μL)	HGB (GM/dL)	HCT (%)	MCV (FL)	MCH (PICO GM)	MCHC (g/dL)	Platelet (1000/μL)	LYMPHUS %	LYMPHUS (1000/μL)	MONOS %	MONOS (1000/μL)	EOSN %	EOSN (1000/μL)	BASOS %
01	1.26	10.73	16.3	57.7	53.7	15.2	28.3	1313	74.9	0.94	0.6	0.01	1.4	0.02	0.4
02	QNS	QNS	QNS	QNS	QNS	QNS	QNS	QNS	QNS	QNS	QNS	QNS	QNS	QNS	QNS
03	1.93	11.59	16.9	61.1	52.8	14.6	27.6	1303	70.5	1.36	0.1	0.00	0.1	0.00	0.2
04	2.86	10.83	16.1	57.7	53.3	14.9	27.9	1419	77.1	2.21	0.2	0.01	0.3	0.01	0.9
05	1.61	10.95	16.2	58.6	53.5	14.8	27.6	1238	71.0	1.15	0.2	0.00	0.7	0.01	0.4
**Mean**	**1.92**	**11.03**	**16.4**	**58.8**	**53.3**	**14.9**	**27.9**	**1318**	**73.4**	**1.42**	**0.3**	**0.01**	**0.6**	**0.01**	**0.5**
**SD**	**0.69**	**0.39**	**0.4**	**1.6**	**0.4**	**0.3**	**0.3**	**75**	**3.2**	**0.56**	**0.2**	**0.01**	**0.6**	**0.01**	**0.3**
**Count**	**4**	**4**	**4**	**4**	**4**	**4**	**4**	**4**	**4**	**4**	**4**	**4**	**4**	**4**	**4**

QNS=Quantity not sufficient

Group: 2 Control Day 14
Males

Animal No.	WBC (1000/μL)	RBC (1000000/μL)	HGB (GM/dL)	HCT (%)	MCV (FL)	MCH (PICO GM)	MCHC (g/dL)	Platelet (1000/μL)	LYMPHUS %	LYMPHUS (1000/μL)	MONOS %	MONOS (1000/μL)	EOSN %	EOSN (1000/μL)	BASOS %
12	4.91	10.05	15.1	55.0	54.7	15.0	27.4	1179	77.4	3.80	0.4	0.02	0.4	0.02	0.4
13	2.57	10.97	16.3	59.3	54.0	14.9	27.6	1621	82.8	2.13	0.3	0.01	0.1	0.00	0.3
14	Clot	Clot	Clot	Clot	Clot	Clot	Clot	Clot	Clot	Clot	Clot	Clot	Clot	Clot	Clot
15	5.20	10.89	16.0	59.3	54.5	14.7	27.0	1632	79.5	4.13	0.6	0.03	0.9	0.05	0.4
41	4.90	10.88	16.1	58.0	53.3	14.8	27.8	1490	85.8	4.20	1.4	0.07	0.6	0.03	0.6
**Mean**	**4.40**	**10.70**	**15.9**	**57.9**	**54.1**	**14.9**	**27.5**	**1481**	**81.4**	**3.57**	**0.7**	**0.03**	**0.5**	**0.03**	**0.4**
**SD**	**1.22**	**0.43**	**0.5**	**2.0**	**0.6**	**0.1**	**0.3**	**211**	**3.7**	**0.97**	**0.5**	**0.03**	**0.3**	**0.02**	**0.1**
**Count**	**4**	**4**	**4**	**4**	**4**	**4**	**4**	**4**	**4**	**4**	**4**	**4**	**4**	**4**	**4**

Group: 3 hEGFRvIII-CD3 bi-scFv Day 2
Males

Animal No.	WBC (1000/μL)	RBC (1000000/μL)	HGB (GM/dL)	HCT (%)	MCV (FL)	MCH (PICO GM)	MCHC (g/dL)	Platelet (1000/μL)	LYMPHUS %	LYMPHUS (1000/μL)	MONOS %	MONOS (1000/μL)	EOSN %	EOSN (1000/μL)	BASOS %
21	1.74	10.91	16.0	57.2	52.4	14.6	27.9	1446	72.3	1.26	0.2	0.00	0.6	0.01	0.3
22	3.57	11.32	16.6	59.9	52.9	14.6	27.6	1408	79.0	2.82	0.2	0.01	0.3	0.01	0.5
23	3.19	11.22	16.4	59.1	52.7	14.7	27.8	1706	74.3	2.37	0.3	0.01	0.4	0.01	0.5
24	5.46	10.99	16.0	59.0	53.7	14.5	27.1	1406	81.9	4.47	0.3	0.02	0.2	0.01	0.8
25	3.53	11.02	16.3	57.7	52.4	14.8	28.2	1301	82.0	2.90	0.3	0.01	0.4	0.01	0.4
**Mean**	**3.50**	**11.09**	**16.3**	**58.6**	**52.8**	**14.6**	**27.7**	**1453**	**77.90**	**2.76**	**0.26**	**0.01**	**0.38**	**0.01**	**0.50**
**SD**	**1.33**	**0.17**	**0.3**	**1.1**	**0.5**	**0.1**	**0.4**	**151**	**4.43**	**1.16**	**0.05**	**0.01**	**0.15**	**0.00**	**0.19**
**Count**	**5**	**5**	**5**	**5**	**5**	**5**	**5**	**5**	**5**	**5**	**5**	**5**	**5**	**5**	**5**

Group: 4 hEGFRvIII-CD3 bi-scFv Day 14
Males

Animal No.	WBC (1000/μL)	RBC (1000000/μL)	HGB (GM/dL)	HCT (%)	MCV (FL)	MCH (PICO GM)	MCHC (g/dL)	Platelet (1000/μL)	LYMPHUS %	LYMPHUS (1000/μL)	MONOS %	MONOS (1000/μL)	EOSN %	EOSN (1000/μL)	BASOS %
31	1.42	10.42	15.6	56.1	53.9	15.0	27.9	1402	72.5	1.03	0.7	0.01	0.3	0.00	0.4
32	3.64	11.89	16.8	61.5	51.7	14.2	27.4	1601	74.4	2.71	0.3	0.01	0.5	0.02	0.4
33	2.23	11.31	16.7	61.1	54.0	14.8	27.3	1412	73.8	1.64	0.5	0.01	1.0	0.02	0.3
34	4.90	10.49	15.1	55.0	52.4	14.4	27.5	1489	68.8	3.37	1.0	0.05	0.5	0.03	0.8
35	5.72	11.18	16.4	60.3	53.9	14.7	27.3	1311	78.1	4.46	1.0	0.06	0.4	0.02	0.5
**Mean**	**3.58**	**11.06**	**16.1**	**58.8**	**53.2**	**14.6**	**27.5**	**1443**	**73.5**	**2.64**	**0.7**	**0.03**	**0.5**	**0.02**	**0.5**
**SD**	**1.79**	**0.61**	**0.7**	**3.0**	**1.1**	**0.3**	**0.2**	**109**	**3.4**	**1.36**	**0.3**	**0.02**	**0.3**	**0.01**	**0.2**
**Count**	**5**	**5**	**5**	**5**	**5**	**5**	**5**	**5**	**5**	**5**	**5**	**5**	**5**	**5**	**5**

Group: 1 Control Day 2
Females

Animal No.	WBC (1000/µL)	RBC (1000000/µL)	HGB (GM/dL)	HCT (%)	MCV (FL)	MCH (PICO GM)	MCHC (g/dL)	Platelet (1000/µL)	LYMPHUS %	LYMPHUS (1000/µL)	MONOS %	MONOS (1000/µL)	EOSN %	EOSN (1000/µL)	BASOS %
06	1.76	10.61	16.0	55.0	51.8	15.1	29.1	1323	55.5	0.98	0.4	0.01	0.3	0.00	0.6
07	3.52	10.63	16.1	56.4	53.0	15.2	28.6	1152	88.4	3.11	0.2	0.01	0.1	0.00	0.4
08	5.06	10.98	16.0	56.8	51.7	14.6	28.2	1322	84.0	4.25	0.3	0.02	0.2	0.01	0.8
09	5.56	11.09	16.9	60.7	54.7	15.2	27.8	1265	82.5	4.59	0.4	0.02	0.3	0.02	0.5
10	1.69	11.34	16.8	59.7	52.6	14.8	28.1	1295	80.3	1.36	0.2	0.00	0.4	0.01	0.7
**Mean**	**3.52**	**10.93**	**16.4**	**57.7**	**52.8**	**15.0**	**28.4**	**1271**	**78.1**	**2.86**	**0.3**	**0.01**	**0.3**	**0.01**	**0.6**
**SD**	**1.80**	**0.31**	**0.5**	**2.4**	**1.2**	**0.3**	**0.5**	**71**	**13.0**	**1.64**	**0.1**	**0.01**	**0.1**	**0.01**	**0.2**
**Count**	**5**	**5**	**5**	**5**	**5**	**5**	**5**	**5**	**5**	**5**	**5**	**5**	**5**	**5**	**5**

Group: 2 Control Day 14
Females

Animal No.	WBC (1000/µL)	RBC (1000000/µL)	HGB (GM/dL)	HCT (%)	MCV (FL)	MCH (PICO GM)	MCHC (g/dL)	Platelet (1000/µL)	LYMPHUS %	LYMPHUS (1000/µL)	MONOS %	MONOS (1000/µL)	EOSN %	EOSN (1000/µL)	BASOS %
16	3.00	10.88	16.2	56.6	52.0	14.9	28.6	1296	75.6	2.27	0.3	0.01	0.4	0.01	0.5
17	2.64	10.90	16.4	57.7	52.9	15.1	28.5	1416	79.7	2.10	0.3	0.01	0.7	0.02	0.9
18	2.41	9.53	14.5	52.0	54.6	15.2	27.9	825	78.0	1.88	0.7	0.02	0.3	0.01	0.5
19	5.92	9.39	14.6	51.3	54.7	15.5	28.4	976	76.5	4.53	0.7	0.04	0.5	0.03	0.4
20	4.77	10.61	15.7	55.7	52.5	14.8	28.2	1127	83.3	3.98	0.9	0.04	0.5	0.02	0.4
**Mean**	**3.75**	**10.26**	**15.5**	**54.7**	**53.3**	**15.1**	**28.3**	**1128**	**78.6**	**2.95**	**0.6**	**0.02**	**0.5**	**0.02**	**0.5**
**SD**	**1.53**	**0.74**	**0.9**	**2.8**	**1.2**	**0.3**	**0.3**	**238**	**3.0**	**1.21**	**0.3**	**0.02**	**0.1**	**0.01**	**0.2**
**Count**	**5**	**5**	**5**	**5**	**5**	**5**	**5**	**5**	**5**	**5**	**5**	**5**	**5**	**5**	**5**

Group: 3 hEGFRvIII-CD3 bi-scFv Day 2
Females

Animal No.	WBC (1000/µL)	RBC (1000000/µL)	HGB (GM/dL)	HCT (%)	MCV (FL)	MCH (PICO GM)	MCHC (g/dL)	Platelet (1000/µL)	LYMPHUS %	LYMPHUS (1000/µL)	MONOS %	MONOS (1000/µL)	EOSN %	EOSN (1000/µL)	BASOS %
26	3.65	10.71	16.0	56.2	52.5	15.0	28.5	1281	87.1	3.18	0.2	0.01	0.5	0.02	1.1
27	QNS	QNS	QNS	QNS	QNS	QNS	QNS	QNS	QNS	QNS	QNS	QNS	QNS	QNS	QNS
28	1.76	10.54	16.0	56.0	53.1	15.2	28.6	1117	73.2	1.29	0.9	0.02	0.1	0.00	0.5
42	4.97	11.21	16.4	58.6	52.3	14.7	28.1	1234	75.3	3.74	0.3	0.02	0.3	0.01	0.4
30	0.73	10.13	15.2	53.5	52.8	15.0	28.5	889	69.4	0.50	0.6	0.00	0.0	0.00	0.2
**Mean**	**2.78**	**10.65**	**15.9**	**56.1**	**52.7**	**15.0**	**28.4**	**1130**	**76.25**	**2.18**	**0.50**	**0.01**	**0.23**	**0.01**	**0.55**
**SD**	**1.90**	**0.45**	**0.5**	**2.1**	**0.4**	**0.2**	**0.2**	**175**	**7.63**	**1.53**	**0.32**	**0.01**	**0.22**	**0.01**	**0.39**
**Count**	**4**	**4**	**4**	**4**	**4**	**4**	**4**	**4**	**4**	**4**	**4**	**4**	**4**	**4**	**4**

QNS=Quantity not sufficient

Group: 4 hEGFRvIII-CD3 bi-scFv Day 14
Females

Animal No.	WBC (1000/µL)	RBC (1000000/µL)	HGB (GM/dL)	HCT (%)	MCV (FL)	MCH (PICO GM)	MCHC (g/dL)	Platelet (1000/µL)	LYMPHUS %	LYMPHUS (1000/µL)	MONOS %	MONOS (1000/µL)	EOSN %	EOSN (1000/µL)	BASOS %
36	3.70	10.80	15.9	57.9	53.6	14.7	27.5	1400	81.0	3.00	0.4	0.01	0.3	0.01	0.5
37	2.44	11.58	17.2	61.3	52.9	14.8	28.1	1405	80.4	1.96	0.3	0.01	1.0	0.03	0.9
38	1.97	10.47	16.0	57.0	54.4	15.2	28.0	1321	85.4	1.68	0.5	0.01	0.9	0.02	0.5
39	4.92	10.64	16.0	56.5	53.1	15.1	28.4	1181	79.7	3.92	1.1	0.06	0.6	0.03	0.7
40	9.68	11.44	16.8	61.3	53.6	14.6	27.3	1247	87.5	8.47	0.9	0.09	0.5	0.05	0.6
**Mean**	**4.54**	**10.99**	**16.4**	**58.8**	**53.5**	**14.9**	**27.9**	**1311**	**82.8**	**3.81**	**0.6**	**0.04**	**0.7**	**0.03**	**0.6**
**SD**	**3.09**	**0.49**	**0.6**	**2.3**	**0.6**	**0.3**	**0.5**	**97**	**3.4**	**2.75**	**0.3**	**0.04**	**0.3**	**0.01**	**0.2**
**Count**	**5**	**5**	**5**	**5**	**5**	**5**	**5**	**5**	**5**	**5**	**5**	**5**	**5**	**5**	**5**

Group: 1 Control Day 2
Males

Animal No.	BUN (mg/dL)	Creatinine (mg/dL)	NA+ (mmol/L)	K+ (mmol/L)	CL- (mmol/L)	ALP (µ/L)	ALT (µ/L)	AST (µ/L)	Total Bilirubin (mg/dL)	GGT (µ/L)	Total Protein (g/dL)	Albumin (g/dL)	Calcium (mg/dL)	PHOS (mg/dL)	Sorbital Dehydrogenase (µ/L)
01	27	0.2	150	9.8	106	210	15	78	QNS	QNS	QNS	3.0	QNS	12.1	QNS
02	31	0.3	152	8.2	112	193	20	60	0.2	QNS	QNS	3.4	0.1	11.9	QNS
03	36	0.3	156	9.3	112	QNS	QNS	22	QNS	QNS	QNS	QNS	QNS	11.4	QNS
04	39	0.3	153	9.0	111	167	22	63	0.2	QNS	QNS	3.3	QNS	9.8	QNS
05	43	0.3	153	9.3	113	181	29	95	QNS	QNS	QNS	3.3	QNS	9.8	QNS
**Mean**	**35**	**0.3**	**153**	**9.1**	**111**	**188**	**22**	**64**	**0.2**			**3.3**	**0.1**	**11.0**	
**SD**	**6**	**0.0**	**2**	**0.6**	**3**	**18**	**6**	**27**	**0.0**			**0.2**		**1.1**	
**Count**	**5**	**5**	**5**	**5**	**5**	**4**	**4**	**5**	**2**	**0**	**0**	**4**	**1**	**5**	**0.0**

QNS=Quantity not sufficient

Group: 2 Control Day 14
Males

Animal No.	BUN (mg/dL)	Creatinine (mg/dL)	NA+ (mmol/L)	K+ (mmol/L)	CL- (mmol/L)	ALP (µ/L)	ALT (µ/L)	AST (µ/L)	Total Bilirubin (mg/dL)	GGT (µ/L)	Total Protein (g/dL)	Albumin (g/dL)	Calcium (mg/dL)	PHOS (mg/dL)	Sorbital Dehydrogenase (µ/L)
12	32	0.3	152	9.7	112	155	18	119	0.2	0	5.2	3.0	10.3	10.8	45.4
13	31	0.3	152	9.7	111	117	24	76	0.2	0	5.3	3.5	10.9	13.6	18.9
14	75	0.3	162	9.2	122	132	52	101	0.2	0	QNS	3.3	9.6	8.6	QNS
15	57	0.3	160	9.7	116	116	30	189	0.2	QNS	QNS	3.1	9.7	10.0	QNS
41	45	0.2	157	>10.0*	116	116	26	131	0.1	0	4.7	3.0	10.0	9.3	27.1
**Mean**	**48**	**0.3**	**157**	**9.6**	**115**	**127**	**30**	**123**	**0.2**	**0**	**5.1**	**3.2**	**10.1**	**10.5**	**30.5**
**SD**	**18**	**0.0**	**5**	**0.3**	**4**	**17**	**13**	**42**	**0.0**	**0**	**0.3**	**0.2**	**0.5**	**1.9**	**13.6**
**Count**	**5**	**5**	**5**	**4**	**5**	**5**	**5**	**5**	**5**	**4**	**3**	**5**	**5**	**5**	**3.0**

QNS=Quantity not sufficient

Group: 3 hEGFRvIII-CD3 bi-scFv Day 2
Males

Animal No.	BUN (mg/dL)	Creatinine (mg/dL)	NA+ (mmol/L)	K+ (mmol/L)	CL- (mmol/L)	ALP (µ/L)	ALT (µ/L)	AST (µ/L)	Total Bilirubin (mg/dL)	GGT (µ/L)	Total Protein (g/dL)	Albumin (g/dL)	Calcium (mg/dL)	PHOS (mg/dL)	Sorbital Dehydrogenase (µ/L)
21	37	0.3	150	8.9	110	155	23	56	0.2	0	QNS	3.3	10.2	9.3	QNS
22	29	0.2	154	7.8	112	124	22	59	0.2	QNS	QNS	3.2	QNS	12.4	QNS
23	34	0.3	154	9.2	114	161	17	68	0.2	QNS	QNS	3.4	QNS	11.4	QNS
24	31	0.2	155	8.6	113	165	17	56	QNS	QNS	QNS	3.2	QNS	12.3	QNS
25	30	0.2	155	9.3	113	QNS	QNS	63	QNS	QNS	QNS	QNS	QNS	9.5	QNS
**Mean**	**32**	**0.2**	**154**	**8.8**	**112**	**151**	**20**	**60**	**0.2**	**0**		**3.3**	**10.2**	**11.0**	
**SD**	**3**	**0.1**	**2**	**0.6**	**2**	**19**	**3**	**5**	**0.0**			**0.1**		**1.5**	
**Count**	**5**	**5**	**5**	**5**	**5**	**4**	**4**	**5**	**3**	**1**	**0**	**4**	**1**	**5**	**0.0**

QNS=Quantity not sufficient

Group: 4 hEGFRvIII-CD3 bi-scFv Day 14
Males

Animal No.	BUN (mg/dL)	Creatinine (mg/dL)	NA+ (mmol/L)	K+ (mmol/L)	CL- (mmol/L)	ALP (µ/L)	ALT (µ/L)	AST (µ/L)	Total Bilirubin (mg/dL)	GGT (µ/L)	Total Protein (g/dL)	Albumin (g/dL)	Calcium (mg/dL)	PHOS (mg/dL)	Sorbital Dehydrogenase (µ/L)
31	74	0.3	155	9.2	115	109	24	79	0.2	0	5.4	3.2	10.3	9.8	47.1
32	34	0.2	156	7.5	113	103	57	143	0.2	0	5.2	3.3	9.6	9.4	49.9
33	60	0.3	161	8.7	116	136	23	94	0.1	0	5.5	3.3	10.5	10.3	49.5
34	41	0.3	158	8.8	114	114	31	99	0.2	0	5.3	3.2	10.2	9.2	48.3
35	39	0.3	157	9.3	114	127	25	118	0.2	0	5.3	3.1	10.5	9.0	41.5
**Mean**	**50**	**0.3**	**157**	**8.7**	**114**	**118**	**32**	**107**	**0.2**	**0**	**5.3**	**3.2**	**10.2**	**9.5**	**47.3**
**SD**	**17**	**0.0**	**2**	**0.7**	**1**	**13**	**14**	**25**	**0.0**	**0**	**0.1**	**0.1**	**0.4**	**0.5**	**3.4**
**Count**	**5**	**5**	**5**	**5**	**5**	**5**	**5**	**5**	**5**	**5**	**5**	**5**	**5**	**5**	**5.0**

***QNS on Rerun of dilution. Not included in analysis.**

Group: 1 Control Day 2
Females

Animal No.	BUN (mg/dL)	Creatinine (mg/dL)	NA+ (mmol/L)	K+ (mmol/L)	CL- (mmol/L)	ALP (µ/L)	ALT (µ/L)	AST (µ/L)	Total Bilirubin (mg/dL)	GGT (µ/L)	Total Protein (g/dL)	Albumin (g/dL)	Calcium (mg/dL)	PHOS (mg/dL)	Sorbital Dehydrogenase (µ/L)
06	31	0.3	154	8.1	115	QNS	QNS	QNS	QNS	QNS	QNS	QNS	QNS	8.9	QNS
07	34	0.3	153	9.7	115	QNS	QNS	78	QNS	QNS	QNS	3.1	QNS	8.7	QNS
08	30	0.3	155	8.5	113	156	31	115	QNS	QNS	QNS	3.3	QNS	10.2	QNS
09	32	0.2	155	9.8	115	QNS	QNS	227	QNS	QNS	QNS	3.5	QNS	11.4	QNS
10	28	0.3	159	7.2	115	QNS	QNS	118	QNS	QNS	QNS	QNS	QNS	9.4	QNS
**Mean**	**31**	**0.3**	**155**	**8.7**	**115**	**156**	**31**	**135**				**3.3**		**9.7**	
**SD**	**2**	**0.0**	**2**	**1.1**	**1**			**64**				**0.2**		**1.1**	
**Count**	**5**	**5**	**5**	**5**	**5**	**1**	**1**	**4**	**0**	**0**	**0**	**3**	**0**	**5**	**0.0**

QNS=Quantity not sufficient

Group: 2 Control Day 14
Females

Animal No.	BUN (mg/dL)	Creatinine (mg/dL)	NA+ (mmol/L)	K+ (mmol/L)	CL- (mmol/L)	ALP (µ/L)	ALT (µ/L)	AST (µ/L)	Total Bilirubin (mg/dL)	GGT (µ/L)	Total Protein (g/dL)	Albumin (g/dL)	Calcium (mg/dL)	PHOS (mg/dL)	Sorbital Dehydrogenase (µ/L)
16	56	0.3	160	7.7	117	89	59	366	QNS	QNS	QNS	3.2	QNS	7.4	QNS
17	52	0.3	155	7.1	114	161	31	176	0.2	0	4.9	3.3	9.7	7.4	QNS
18	40	0.2	157	9.1	112	177	34	195	0.2	0	4.8	2.9	9.6	8.5	44.4
19	33	0.2	154	9.1	111	166	35	184	0.3	0	4.7	2.9	9.7	8.8	48.7
20	60	0.2	157	8.5	115	151	71	259	0.2	0	4.4	3.0	9.6	8.4	QNS
**Mean**	**48**	**0.2**	**157**	**8.3**	**114**	**149**	**46**	**236**	**0.2**	**0**	**4.7**	**3.1**	**9.7**	**8.1**	**46.6**
**SD**	**11**	**0.1**	**2**	**0.9**	**2**	**35**	**18**	**80**	**0.1**	**0**	**0.2**	**0.2**	**0.1**	**0.7**	**3.0**
**Count**	**5**	**5**	**5**	**5**	**5**	**5**	**5**	**5**	**4**	**4**	**4**	**5**	**4**	**5**	**2.0**

QNS=Quantity not sufficient

Group: 3 hEGFRvIII-CD3 bi-scFv Day 2
Females

Animal No.	BUN (mg/dL)	Creatinine (mg/dL)	NA+ (mmol/L)	K+ (mmol/L)	CL- (mmol/L)	ALP (µ/L)	ALT (µ/L)	AST (µ/L)	Total Bilirubin (mg/dL)	GGT (µ/L)	Total Protein (g/dL)	Albumin (g/dL)	Calcium (mg/dL)	PHOS (mg/dL)	Sorbital Dehydrogenase (µ/L)
26	35	0.3	152	8.2	109	QNS	QNS	68	QNS	QNS	QNS	0.0	QNS	8.9	QNS
27	25	QNSR	154	8.7	109	QNS	QNS	QNS	QNS	QNS	QNS	QNS	QNS	10.5	QNS
28	36	0.3	154	QNSR	114	174	QNS	278	QNS	QNS	QNS	3.3	QNS	10.0	QNS
42	25	0.2	156	7.9	114	QNS	QNS	69	QNS	QNS	QNS	QNS	QNS	9.3	QNS
30	29	0.2	154	8.3	108	269	26	233	QNS	QNS	QNS	3.2	QNS	10.5	QNS
**Mean**	**30**	**0.3**	**154**	**8.3**	**111**	**222**	**26**	**162**				**2.2**		**9.8**	
**SD**	**5**	**0.1**	**1**	**0.3**	**3**	**67**		**110**				**1.9**		**0.7**	
**Count**	**5**	**4**	**5**	**4**	**5**	**2**	**1**	**4**	**0**	**0**	**0**	**3**	**0**	**5**	**0.0**

QNS=Quantity not sufficient; QNSR=Quantity not sufficient to repeat

Group: 4 hEGFRvIII-CD3 bi-scFv Day 14
Females

Animal No.	BUN (mg/dL)	Creatinine (mg/dL)	NA+ (mmol/L)	K+ (mmol/L)	CL- (mmol/L)	ALP (µ/L)	ALT (µ/L)	AST (µ/L)	Total Bilirubin (mg/dL)	GGT (µ/L)	Total Protein (g/dL)	Albumin (g/dL)	Calcium (mg/dL)	PHOS (mg/dL)	Sorbital Dehydrogenase (µ/L)
36	49	0.3	156	8.2	116	174	25	169	0.2	0	5.3	3.3	10.2	9.6	36.9
37	65	0.3	157	9.7	117	191	35	289	0.2	0	5.0	3.3	10.0	9.5	QNS
38	68	0.3	154	8.4	110	195	35	148	0.3	0	5.4	3.4	9.9	9.3	47.0
39	45	0.2	158	8.4	118	174	47	214	0.2	QNS	QNS	3.0	QNS	9.1	QNS
40	25	<0.2	158	9.0	119	119	36	280	QNS	QNS	QNS	3.3	QNS	9.6	QNS
**Mean**	**50**	**0.3**	**157**	**8.7**	**116**	**171**	**36**	**220**	**0.2**	**0**	**5.2**	**3.3**	**10.0**	**9.4**	**42.0**
**SD**	**17**	**0.1**	**2**	**0.6**	**4**	**30**	**8**	**64**	**0.1**	**0**	**0.2**	**0.2**	**0.2**	**0.2**	**7.1**
**Count**	**5**	**4**	**5**	**5**	**5**	**5**	**5**	**5**	**4**	**3**	**3**	**5**	**3**	**5**	**2.0**

QNS=Quantity not sufficient

Group: 1 Control Day 2
Males

Animal No.	Color	Clarity	Glucose (mg/dL)	Ketones (mg/dL)	Blood (# Plus)	Protein (mg/dL)	Bilirubin (# Plus)	pH (pH Units)	Urobilinogen (mg/dL)	Specific gravity (SG units)	Total Volume MLS	Bacteria / HPF
01	Yellow	Clear	Neg	Neg	Neg	Neg	Neg	6.0	0.2	1.024	1.7	NS
02	Straw	Clear	Neg	Neg	Neg	Neg	Neg	7.0	0.2	1.020	1.0	NS
03	Yellow	Clear	Neg	Neg	Neg	Trace	Neg	6.5	0.2	1.040	1.1	NS
04	Yellow	Clear	Neg	Neg	Neg	Neg	Neg	6.5	0.2	1.024	1.3	NS
05	Yellow	Slightly Cloudy	Neg	Trace	Neg	Neg	Neg	6.5	0.2	1.027	1.6	NS
**Mean**	-	-	-	-	-	-	-	**6.5**	**0.2**	**1.027**	**1.3**	-
**SD**	-	-	-	-	-	-	-	**0.4**	**0.0**	**0.008**	**0.3**	-
**Count**	-	-	-	-	-	-	-	**5**	**5**	**5**	**5**	-

Neg=Negative; NS=None seen

Group: 2 Control Day 14
Males

Animal No.	Color	Clarity	Glucose (mg/dL)	Ketones (mg/dL)	Blood (# Plus)	Protein (mg/dL)	Bilirubin (# Plus)	pH (pH Units)	Urobilinogen (mg/dL)	Specific gravity (SG units)	Total Volume MLS	Bacteria / HPF
12	Yellow	Clear	Neg	Neg	Neg	Neg	Neg	6.0	0.2	1.028	0.6	Few
13	Yellow	Clear	Neg	Trace	Neg	Neg	Neg	6.5	0.2	1.026	1.2	Moderate
14	Yellow	Clear	Neg	Neg	Neg	Neg	Neg	7.0	0.2	1.026	1.4	Few
15	Yellow	Clear	Neg	Trace	Neg	Trace	Neg	7.0	0.2	1.032	0.9	Moderate
41	Yellow	Clear	Neg	Neg	Neg	Neg	Neg	7.0	0.2	1.022	1.2	Moderate
**Mean**	-	-	-	-	-	-	-	**6.7**	**0.2**	**1.027**	**1.1**	-
**SD**	-	-	-	-	-	-	-	**0.4**	**0.0**	**0.004**	**0.3**	-
**Count**	**10**	**10**	-	-	-	-	-	**5**	**5**	**5**	**5**	-

Neg=Negative; NS=None seen; MOAP=Moderate Amorphous; MAAP=Many Amorphous

Group: 3 hEGFRvIII-CD3 bi-scFv Day 2
Males

Animal No.	Color	Clarity	Glucose (mg/dL)	Ketones (mg/dL)	Blood (# Plus)	Protein (mg/dL)	Bilirubin (# Plus)	pH (pH Units)	Urobilinogen (mg/dL)	Specific gravity (SG units)	Total Volume MLS	Bacteria / HPF
21	Yellow	Clear	Neg	Trace	Neg	Neg	Neg	8.0	0.2	1.020	1.2	NS
22	Yellow	Clear	Neg	Neg	Neg	Neg	Neg	7.0	0.2	1.036	0.9	NS
23	Yellow	Clear	Neg	Neg	Neg	Neg	Neg	7.0	0.2	1.024	0.9	NS
24	Yellow	Clear	Neg	Neg	Neg	Neg	Neg	7.0	0.2	1.023	1.8	NS
25	Yellow	Clear	Neg	Neg	Neg	Neg	Neg	6.5	0.2	1.026	1.5	NS
**Mean**	-	-	-	-	-	-	-	**7.1**	**0.2**	**1.026**	**1.3**	-
**SD**	-	-	-	-	-	-	-	**0.5**	**0.0**	**0.006**	**0.4**	-
**Count**	**10**	**10**	-	-	-	-	-	**5**	**5**	**5**	**5**	-

Neg=Negative; NS=None seen

Group: 4 hEGFRvIII-CD3 bi-scFv Day 14
Males

Animal No.	Yellow	Clarity	Glucose (mg/dL)	Ketones (mg/dL)	Blood (# Plus)	Protein (mg/dL)	Bilirubin (# Plus)	pH (pH Units)	Urobilinogen (mg/dL)	Specific gravity (SG units)	Total Volume MLS	Bacteria / HPF
31	Yellow	Clear	Neg	Trace	Neg	Trace	Neg	6.5	0.2	1.020	1.6	Few
32	Yellow	Clear	Neg	Trace	Neg	Trace	Neg	6.5	0.2	QNS	0.3	Few
33	Yellow	Slightly Cloudly	Neg	Trace	Small	30.0	Neg	7.0	0.2	1.022	1.8	Few
34	Yellow	Clear	Neg	Trace	Neg	Trace	Neg	7.0	0.2	1.018	1.0	Few
35	Yellow	Clear	Neg	Trace	Neg	Trace	Neg	6.5	0.2	1.031	1.5	Few
**Mean**						-		**7.1**	**0.2**	**1.026**	**1.3**	
**SD**	-	-	-	-	-	-	-	**0.3**	**0.0**	**0.006**	**0.6**	-
**Count**	**10**	**10**	-	-	-	-	-	**5**	**5**	**4**	**5**	-

Neg=Negative; NS=None seen; MOAP=Moderate Amorphous

Group: 1 Control Day 2
Males

Animal No.	Bacteria / HPF	Urine WBC	Urine RBC	Epithelial Cells	Calcium Oxalate Crystals	Triple Phosphate Crystals	Mucus	Yeast	Other Findings
01	NS	Few	NS	NS	NS	NS	NS	NS	NS
02	NS	Few	NS	NS	NS	NS	NS	NS	NS
03	NS	Few	NS	NS	NS	NS	NS	NS	NS
04	NS	Few	NS	NS	NS	NS	NS	NS	NS
05	NS	Few	NS	NS	NS	NS	NS	NS	NS
**Mean**	-	-	-	-	-	-	-	-	-
**SD**	-	-	-	-	-	-	-	-	-
**Count**	-	-	-	-	-	-	-	-	-

Neg=Negative; NS=None seen

Group: 2 Control Day 14
Males

Animal No.	Bacteria / HPF	Urine WBC	Urine RBC	Epithelial Cells	Calcium Oxalate Crystals	Triple Phosphate Crystals	Mucus	Yeast	Other Findings
12	Few	Few	NS	NS	NS	NS	NS	NS	NS
13	Moderate	Few	NS	NS	NS	NS	NS	NS	MOAP
14	Few	Few	NS	NS	NS	NS	NS	NS	MOAP
15	Moderate	Few	NS	NS	NS	NS	NS	NS	MAAP
41	Moderate	Few	NS	NS	NS	NS	NS	NS	MAAP
**Mean**	-	-	-	-	-	-	-	-	-
**SD**	-	-	-	-	-	-	-	-	-
**Count**	-	-	-	-	-	-	-	-	-

Neg=Negative; NS=None seen; MOAP=Moderate Amorphous; MAAP=Many Amorphous

Group: 3 hEGFRvIII-CD3 bi-scFv Day 2
Males

Animal No.	Bacteria / HPF	Urine WBC	Urine RBC	Epithelial Cells	Calcium Oxalate Crystals	Triple Phosphate Crystals	Mucus	Yeast	Other Findings
21	NS	NS	NS	NS	NS	Few	NS	NS	NS
22	NS	Few	NS	NS	NS	NS	NS	NS	NS
23	NS	NS	NS	NS	NS	Few	NS	NS	NS
24	NS	NS	NS	NS	NS	NS	NS	NS	NS
25	NS	NS	NS	NS	NS	NS	NS	NS	NS
**Mean**	-	-	-	-	-	-	-	-	-
**SD**	-	-	-	-	-	-	-	-	-
**Count**	-	-	-	-	-	-	-	-	-

Neg=Negative; NS=None seen

Group: 4 hEGFRvIII-CD3 bi-scFv Day 14
Males

Animal No.	Bacteria / HPF	Urine WBC	Urine RBC	Epithelial Cells	Calcium Oxalate Crystals	Triple Phosphate Crystals	Mucus	Yeast	Other Findings
31	Few	Few	NS	NS	NS	NS	NS	NS	NS
32	Few	Few	NS	NS	NS	NS	NS	NS	MOAP
33	Few	NS	NS	NS	NS	NS	NS	NS	NS
34	Few	NS	NS	NS	NS	NS	NS	NS	NS
35	Few	NS	NS	NS	NS	NS	NS	NS	NS
**Mean**									
**SD**	-	-	-	-	-	-	-	-	-
**Count**	-	-	-	-	-	-	-	-	-

Neg=Negative; NS=None seen; MOAP=Moderate Amorphous

Group: 1 Control Day 2
Females

Animal No.	Color	Clarity	Glucose (mg/dL)	Ketones (mg/dL)	Blood (# Plus)	Protein (mg/dL)	Bilirubin (# Plus)	pH (pH Units)	Urobilinogen (mg/dL)	Specific gravity (SG units)	Total Volume MLS
06											
07	Yellow	Clear	Neg	Trace	Neg	Neg	Neg	7.0	0.2	QNS	0.2
08	Yellow	Clear	Neg	Trace	Neg	Neg	Neg	6.5	0.2	1.026	0.6
09	Yellow	Clear	Neg	Trace	Neg	Trace	Neg	6.5	0.2	1.042	0.4
10	Yellow	Clear	Neg	Neg	Neg	Neg	Neg	7.0	0.2	1.023	1.1
**Mean**	-	-	-	-	-	-	-	**6.8**	**0.2**	**1.030**	**0.6**
**SD**	-	-	-	-	-	-	-	**0.3**	**0.0**	**0.010**	**0.4**
**Count**	-	-	-	-	-	-	-	**4**	**4**	**3**	**4**

Neg=Negative; QNS=Quantity not sufficient; NS=None seen

Group: 2 Control Day 14
Females

Animal No.	Color	Clarity	Glucose (mg/dL)	Ketones (mg/dL)	Blood (# Plus)	Protein (mg/dL)	Bilirubin (# Plus)	pH (pH Units)	Urobilinogen (mg/dL)	Specific gravity (SG units)	Total Volume MLS
16	QNS	QNS	QNS	QNS	QNS	QNS	QNS	QNS	QNS	QNS	0.0
17	Yellow	Clear	Neg	Trace	Neg	Neg	Neg	7.0	0.2	1.024	0.4
18	Yellow	Clear	Neg	Trace	Neg	Neg	Neg	6.0	0.2	1.026	1.0
19	Dark Yellow	Clear	Neg	Trace	Neg	Trace	Small	5.5	1.0	1.050	0.3
20	Yellow	Clear	Neg	Trace	Neg	Neg	Neg	7.0	0.2	1.015	1.8
**Mean**	-	-	-	-	-	-	-	**6.4**	**0.4**	**1.029**	**0.7**
**SD**	-	-	-	-	-	-	-	**0.8**	**0.4**	**0.015**	**0.7**
**Count**	**10**	**10**	-	-	-	-	-	**4**	**4**	**4**	**5**

Neg=Negative; QNS=Quantity not sufficient; NS=None seen; MAAP=Many Amorphous

Group: 3 hEGFRvIII-CD3 bi-scFv Day 2
Females

Animal No.	Color	Clarity	Glucose (mg/dL)	Ketones (mg/dL)	Blood (# Plus)	Protein (mg/dL)	Bilirubin (# Plus)	pH (pH Units)	Urobilinogen (mg/dL)	Specific gravity (SG units)	Total Volume MLS
26	Yellow	Clear	Neg	Neg	Neg	Neg	Neg	7.0	0.2	1.027	0.4
27	Yellow	Clear	Neg	Trace	Neg	Trace	Neg	6.0	0.2	QNS	0.2
28	Yellow	Clear	Neg	Trace	Neg	Neg	Neg	6.5	0.2	1.021	0.8
42	Yellow	Clear	Neg	Trace	Neg	Neg	Neg	7.0	0.2	QNS	0.2
30	Yellow	Clear	Neg	Neg	Neg	Neg	Neg	5.5	0.2	1.026	0.8
**Mean**	-	-	-	-	-	-	-	**6.4**	**0.2**	**1.025**	**0.5**
**SD**	-	-	-	-	-	-	-	**0.7**	**0.0**	**0.003**	**0.3**
**Count**	**10**	**10**	-	-	-	-	-	**5**	**5**	**3**	**5**

Neg=Negative; QNS=Quantity not sufficient; NS=None seen

Group: 4 hEGFRvIII-CD3 bi-scFv Day 14
Females

Animal No.	Color	Clarity	Glucose (mg/dL)	Ketones (mg/dL)	Blood (# Plus)	Protein (mg/dL)	Bilirubin (# Plus)	pH (pH Units)	Urobilinogen (mg/dL)	Specific gravity (SG units)	Total Volume MLS
36	Yellow	Clear	Neg	Trace	Neg	30	Neg	7.0	0.2	1.035	0.5
37	Yellow	Clear	Neg	15.0	Neg	Trace	Neg	6.0	0.2	1.031	1.0
38	Yellow	Clear	Neg	Trace	Neg	Trace	Neg	6.5	0.2	1.030	1.4
39	Straw	Clear	Neg	Neg	Neg	Neg	Neg	6.0	0.2	1.008	5.6
40	Yellow	Clear	Neg	15.0	Neg	Trace	Neg	7.0	0.2	QNS	0.2
**Mean**						-		**6.5**	**0.2**	**1.026**	**1.7**
**SD**	-	-	-	-	-	-	-	**0.5**	**0.0**	**0.012**	**2.0**
**Count**	**10**	**10**	-	-	-	-	-	**5**	**5**	**4**	**6**

Neg=Negative; QNS=Quantity not sufficient; NS=None seen

Group: 1 Control Day 2
Females

Animal No.	Bacteria / HPF	Urine WBC	Urine RBC	Epithelial Cells	Calcium Oxalate Crystals	Triple Phosphate Crystals	Mucus	Yeast	Other Findings
06									
07	NS	NS	NS	NS	NS	NS	NS	NS	NS
08	NS	NS	NS	NS	NS	NS	NS	NS	NS
09	NS	NS	NS	NS	NS	NS	NS	NS	NS
10	NS	NS	NS	NS	NS	NS	NS	NS	NS
**Mean**	-	-	-	-	-	-	-	-	-
**SD**	-	-	-	-	-	-	-	-	-
**Count**	-	-	-	-	-	-	-	-	-

Neg=Negative; QNS=Quantity not sufficient; NS=None seen

Group: 2 Control Day 14
Females

Animal No.	Bacteria / HPF	Urine WBC	Urine RBC	Epithelial Cells	Calcium Oxalate Crystals	Triple Phosphate Crystals	Mucus	Yeast	Other Findings
16	QNS	QNS	QNS	QNS	QNS	QNS	QNS	QNS	QNS
17	NS	NS	NS	NS	NS	NS	NS	NS	NS
18	Few	Moderate	NS	NS	NS	NS	NS	NS	NS
19	Few	Few	NS	NS	NS	NS	NS	NS	MAAP
20	Few	Few	NS	NS	NS	NS	NS	NS	NS
**Mean**	-	-	-	-	-	-	-	-	-
**SD**	-	-	-	-	-	-	-	-	-
**Count**	-	-	-	-	-	-	-	-	-

Neg=Negative; QNS=Quantity not sufficient; NS=None seen; MAAP=Many Amorphous

Group: 3 hEGFRvIII-CD3 bi-scFv Day 2
Females

Animal No.	Bacteria / HPF	Urine WBC	Urine RBC	Epithelial Cells	Calcium Oxalate Crystals	Triple Phosphate Crystals	Mucus	Yeast	Other Findings
26	NS	NS	NS	NS	NS	NS	NS	NS	NS
27	NS	NS	NS	NS	NS	NS	NS	NS	NS
28	NS	NS	NS	NS	NS	NS	NS	NS	NS
42	NS	NS	NS	NS	NS	NS	NS	NS	NS
30	NS	NS	NS	NS	NS	NS	NS	NS	NS
**Mean**	-	-	-	-	-	-	-	-	-
**SD**	-	-	-	-	-	-	-	-	-
**Count**	-	-	-	-	-	-	-	-	-

Neg=Negative; QNS=Quantity not sufficient; NS=None seen

Group: 4 hEGFRvIII-CD3 bi-scFv Day 14
Females

Animal No.	Bacteria / HPF	Urine WBC	Urine RBC	Epithelial Cells	Calcium Oxalate Crystals	Triple Phosphate Crystals	Mucus	Yeast	Other Findings
36	Few	NS	NS	NS	NS	NS	NS	NS	NS
37	Few	NS	NS	NS	NS	NS	NS	NS	NS
38	Few	NS	NS	NS	NS	NS	NS	NS	NS
39	Many	NS	NS	NS	NS	NS	NS	NS	NS
40	QNS	QNS	QNS	QNS	QNS	QNS	QNS	QNS	QNS
**Mean**									
**SD**	-	-	-	-	-	-	-	-	-
**Count**	-	-	-	-	-	-	-	-	-

Neg=Negative; QNS=Quantity not sufficient; NS=None seen

S2 Table – Intergroup comparison of gross and histopathological observations for day 2 group animals.

	MALES		FEMALES	
	Control	hEGFRvII	Control	hEGFRvII
Number of Animals on Study :	5	5	5	5
Number of Animals Completed:	(5)	(5)	(5)	(5)
BRAIN;				
Examined	(5)	(5)	(5)	(5)
Within Normal Limits	5	5	4	5
Cyst; squamous	0	0	1	0
HEART;				
Examined	(5)	(5)	(5)	(5)
Within Normal Limits	5	5	5	5
KIDNEYS;				
Examined	(5)	(5)	(5)	(5)
Within Normal Limits	5	5	5	4
Cast; proteinacious	(0)	(0)	(0)	(1)
minimal	0	0	0	1
LIVER;				
Examined	(5)	(5)	(5)	(5)
Within Normal Limits	5	5	5	4
Necrosis; multifocal	(0)	(0)	(0)	(1)
minimal	0	0	0	1
LUNGS;				
Examined	(5)	(5)	(5)	(5)
Within Normal Limits	5	5	5	5
LYMPH NODE, INGUINAL;				
Examined	(5)	(5)	(5)	(5)
Within Normal Limits	5	5	5	5
LYMPH NODE, MESENTERIC;				
Examined	(5)	(5)	(5)	(5)
Within Normal Limits	2	0	1	0
Apoptosis; lymphocytic	(3)	(5)	(4)	(5)
minimal	1	2	1	5
mild	2	2	3	0
moderate	0	1	0	0
SPLEEN;				
Examined	(5)	(5)	(5)	(5)
Within Normal Limits	4	2	4	1
Hematopoiesis; increased	(1)	(1)	(0)	(1)
minimal	1	1	0	1
Pigmentation; melanin	(0)	(2)	(1)	(3)
minimal	0	1	0	3
mild	0	1	1	0
THYMUS;				
Examined	(5)	(5)	(5)	(5)
Within Normal Limits	0	0	0	0
Cyst	3	4	3	1
Hyperplasia; atypical	(5)	(5)	(5)	(5)
moderate	5	5	5	5

S3 Table – Intergroup comparison of gross and histopathological observations for day 14 group animals.

	MALES		FEMALES	
	Control	hEGFRvII	Control	hEGFRvII
Number of Animals on Study :	5	5	5	5
Number of Animals Completed:	(5)	(5)	(5)	(5)
BRAIN;				
Examined	(5)	(5)	(5)	(5)
Within Normal Limits	5	5	5	5
HEART;				
Examined	(5)	(5)	(5)	(5)
Within Normal Limits	5	5	5	5
KIDNEYS;				
Examined	(5)	(5)	(5)	(5)
Within Normal Limits	5	5	4	4
Degeneration; tubule; focal	(0)	(0)	(0)	(1)
minimal	0	0	0	1
Dilation; pelvis	(0)	(0)	(1)	(0)
mild	0	0	1	0
LIVER;				
Examined	(5)	(5)	(5)	(5)
Within Normal Limits	4	4	4	5
Infiltration; mixed; focal	(0)	(1)	(1)	(0)
minimal	0	1	1	0
Accumulation; glycogen; hepatocyte; focal	(1)	(0)	(0)	(0)
mild	1	0	0	0
LUNGS;				
Examined	(5)	(5)	(5)	(5)
Within Normal Limits	5	5	5	5
LYMPH NODE, INGUINAL;				
Examined	(5)	(5)	(5)	(5)
Within Normal Limits	5	5	5	5
LYMPH NODE, MESENTERIC;				
Examined	(5)	(5)	(5)	(5)
Within Normal Limits	2	1	1	1
Histocytosis	(0)	(0)	(1)	(0)
mild	0	0	1	0
Apoptosis; lymphocytic	(3)	(4)	(4)	(4)
minimal	1	3	2	3
mild	2	1	2	1
NO CORRELATING LESION;				
Examined	(0)	(0)	(0)	(1)
Within Normal Limits	0	0	0	0
No Microscopic Correlation	0	0	0	1
SPLEEN;				
Examined	(5)	(5)	(5)	(5)
Within Normal Limits	5	3	1	4
Hematopoiesis; increased	(0)	(0)	(1)	(1)
minimal	0	0	1	1
Pigmentation; melanin	(0)	(2)	(3)	(0)
minimal	0	0	1	0
mild	0	2	2	0
THYMUS;				
Examined	(5)	(5)	(5)	(5)
Within Normal Limits	1	0	1	0
Cyst	2	4	2	1
Hyperplasia; atypical	(4)	(5)	(4)	(5)
mild	3	2	0	2
moderate	1	3	4	3
Ectopia	0	0	1	0

S4 Table – Aggregated day 2 p-value tables

hEGFRvIII-CD3 bi-scFv Exposure Level	Sex	Final Body Weight	Body Weight Gain	Food Consumption	EAE Observations
0 mg/kg	M	-	-	-	-
7.5 mg/kg		0.8363	0.8084	0.7829	1.0000

hEGFRvIII-CD3 bi-scFv Exposure Level	Sex	Final Body Weight	Body Weight Gain	Food Consumption	EAE Observations
0 mg/kg	F	-	-	-	-
7.5 mg/kg		0.9504	0.7714	0.4270	1.0000

hEGFRvIII-CD3 bi-scFv Exposure Level	Sex	Specific Gravity	Bilirubin	Ketones	Blood	pH	Protein	Total Volume	Glucose	Urobilinogen
0 mg/kg	M	-	-	-	-	-	-	-	-	-
7.5 mg/kg		0.7913	1.0000	1.0000	1.0000	0.0736	0.3466	0.7277	1.0000	1.0000

hEGFRvIII-CD3 bi-scFv Exposure Level	Sex	Specific Gravity	Bilirubin	Ketones	Blood	pH	Protein	Total Volume	Glucose	Urobilinogen
0 mg/kg	F	-	-	-	-	-	-	-	-	-
7.5 mg/kg		0.4112	1.0000	0.6845	1.0000	0.3558	0.8786	0.9335	1.0000	1.0000

hEGFRvIII-CD3 bi-scFv Exposure Level	Sex	White Blood Cell	Red Blood Cell	Hemaglobin	Hematocrit	Mean Corpuscular Volume	Mean Corpuscular Hemaglobin	Mean Corpuscular Hemaglobin Co	Platelets	% Lymphocytes	Lymphocytes
0 mg/kg	M	-	-	-	-	-	-	-	-	-	-
7.5 mg/kg		0.0689	0.7360	0.5939	0.8347	0.1588	0.1001	0.6236	0.1492	0.1303	0.0714

hEGFRvIII-CD3 bi-scFv Exposure Level	Sex	% Monocytes	Monocytes	% Eosinophils	Eosinophils	% Basophils	Basophils	% Large Unstained Cells	Large Unstained Cells	% Neutrophils	Neutrophils
0 mg/kg	M	-	-	-	-	-	-	-	-	-	-
7.5 mg/kg		0.8864	0.2924	0.3825	1.0000	0.8816	0.3269	0.6239	0.4853	0.3303	0.0721

hEGFRvIII-CD3 bi-scFv Exposure Level	Sex	Sodium	Potassium	Chloride	Calcium	Phosphorous	Aspartate aminotransferase	Alanine aminotransferase	Alkaline phosphatase	Gamma glutamyl transferase
0 mg/kg	M	-	-	-	-	-	-	-	-	-
7.5 mg/kg		0.5675	0.3674	0.2907	QNS	0.9815	0.8018	0.6164	0.0312	QNS

hEGFRvIII-CD3 bi-scFv Exposure Level	Sex	Blood urea nitrogen	Creatinine	Total Bilirubin	Sorbitol dehydrogenase	Total Protein	Albumin
0 mg/kg	M	-	-	-	-	-	-
7.5 mg/kg		0.3746	0.2415	QNS	QNS	QNS	0.8090

hEGFRvIII-CD3 bi-scFv Exposure Level	Sex	White Blood Cell	Red Blood Cell	Hemaglobin	Hematocrit	Mean Corpuscular Volume	Mean Corpuscular Hemaglobin	Mean Corpuscular Hemaglobin Co	Platelets	% Lymphocytes	Lymphocytes
0 mg/kg	F	-	-	-	-	-	-	-	-	-	-
7.5 mg/kg		0.5681	0.2994	0.1911	0.3142	0.8972	0.9764	0.8186	0.1401	0.8056	0.5451

hEGFRvIII-CD3 bi-scFv Exposure Level	Sex	% Monocytes	Monocytes	% Eosinophils	Eosinophils	% Basophils	Basophils	% Large Unstained Cells	Large Unstained Cells	% Neutrophils	Neutrophils
0 mg/kg	F	-	-	-	-	-	-	-	-	-	-
7.5 mg/kg		0.2182	0.9356	0.7663	0.9356	0.7980	0.8165	0.4561	0.9454	0.8734	0.7908

hEGFRvIII-CD3 bi-scFv Exposure Level	Sex	Sodium	Potassium	Chloride	Calcium	Phosphorous	Aspartate aminotransferase	Alanine aminotransferase	Alkaline phosphatase	Gamma glutamyl transferase
0 mg/kg	F	-	-	-	-	-	-	-	-	-
7.5 mg/kg		0.3466	0.5260	*	QNS	0.8437	0.6801	QNS	0.5719	QNS

hEGFRvIII-CD3 bi-scFv Exposure Level	Sex	Blood urea nitrogen	Creatinine	Total Bilirubin	Sorbitol dehydrogenase	Total Protein	Albumin
0 mg/kg	F	-	-	-	-	-	-
7.5 mg/kg		^	0.4071	QNS	QNS	QNS	0.3571

^Hetergeneous data set with no pair-wise differences found. No p-values are given for non-parametric Dunn's test
*Hetergeneous data set with significant pair-wise differences found. No p-values are given for non-parametric Dunn's test
QNS=Quantity not sufficient

S5 Table – Aggregated day 14 p-value tables

hEGFRvIII-CD3 bi-scFv Exposure Level	Sex	Final Body Weight	Body Weight Gain	Food Consumption	EAE Observations
0 mg/kg	M	-	-	-	-
7.5 mg/kg		0.5830	0.6926	0.7072	1.0000

hEGFRvIII-CD3 bi-scFv Exposure Level	Sex	Final Body Weight	Body Weight Gain	Food Consumption	EAE Observations
0 mg/kg	F	-	-	-	-
7.5 mg/kg		0.7985	0.6760	0.4722	1.0000

hEGFRvIII-CD3 bi-scFv Exposure Level	Sex	Specific Gravity	Bilirubin	Ketones	Blood	pH	Protein	Total Volume	Glucose	Urobilinogen
0 mg/kg	M	-	-	-	-	-	-	-	-	-
7.5 mg/kg		0.2356	1.0000	^	0.3466	1.0000	0.0034	0.5697	1.0000	1.0000

hEGFRvIII-CD3 bi-scFv Exposure Level	Sex	Specific Gravity	Bilirubin	Ketones	Blood	pH	Protein	Total Volume	Glucose	Urobilinogen
0 mg/kg	F	-	-	-	-	-	-	-	-	-
7.5 mg/kg		0.7852	0.2924	0.8046	1.0000	0.7724	0.1099	0.4798	1.0000	0.2924

hEGFRvIII-CD3 bi-scFv Exposure Level	Sex	White Blood Cell	Red Blood Cell	Hemaglobin	Hematocrit	Mean Corpuscular Volume	Mean Corpuscular Hemaglobin	Mean Corpuscular Hemaglobin Co	Platelets	% Lymphocytes	Lymphocytes
0 mg/kg	M	-	-	-	-	-	-	-	-	-	-
7.5 mg/kg		0.4661	0.3553	0.5964	0.6273	0.1617	0.2220	0.8827	0.7382	0.0124	0.2936

hEGFRvIII-CD3 bi-scFv Exposure Level	Sex	% Monocytes	Monocytes	% Eosinophils	Eosinophils	% Basophils	Basophils	% Large Unstained Cells	Large Unstained Cells	% Neutrophils	Neutrophils
0 mg/kg	M	-	-	-	-	-	-	-	-	-	-
7.5 mg/kg		0.9286	0.8001	0.8483	0.5338	0.6387	1.0000	0.4389	0.4601	0.0701	0.8982

hEGFRvIII-CD3 bi-scFv Exposure Level	Sex	Sodium	Potassium	Chloride	Calcium	Phosphorous	Aspartate aminotransferase	Alanine aminotransferase	Alkaline phosphatase	Gamma glutamyl transferase
0 mg/kg	M	-	-	-	-	-	-	-	-	-
7.5 mg/kg		0.7353	0.0548	0.6314	0.6869	0.3347	0.4695	0.8232	0.3604	1.0000

hEGFRvIII-CD3 bi-scFv Exposure Level	Sex	Blood urea nitrogen	Creatinine	Total Bilirubin	Sorbitol dehydrogenase	Total Protein	Albumin
0 mg/kg	M	-	-	-	-	-	-
7.5 mg/kg		0.8897	1.0000	1.0000	*	0.1215	0.7103

hEGFRvIII-CD3 bi-scFv Exposure Level	Sex	White Blood Cell	Red Blood Cell	Hemaglobin	Hematocrit	Mean Corpuscular Volume	Mean Corpuscular Hemaglobin	Mean Corpuscular Hemaglobin Co	Platelets	% Lymphocytes	Lymphocytes
0 mg/kg	F	-	-	-	-	-	-	-	-	-	-
7.5 mg/kg		0.6208	0.1072	0.0948	0.0362	^	0.2280	0.0878	0.1502	0.0765	0.5435

hEGFRvIII-CD3 bi-scFv Exposure Level	Sex	% Monocytes	Monocytes	% Eosinophils	Eosinophils	% Basophils	Basophils	% Large Unstained Cells	Large Unstained Cells	% Neutrophils	Neutrophils
0 mg/kg	F	-	-	-	-	-	-	-	-	-	-
7.5mg/kg		0.7661	0.5225	0.2494	0.2256	0.4257	0.4082	0.6276	0.6286	0.0871	0.5723

hEGFRvIII-CD3 bi-scFv Exposure Level	Sex	Sodium	Potassium	Chloride	Calcium	Phosphorous	Aspartate aminotransferase	Alanine aminotransferase	Alkaline phosphatase	Gamma glutamyl transferase
0 mg/kg	F	-	-	-	-	-	-	-	-	-
7.5 mg/kg		1.0000	0.3873	0.2822	0.0053	*	0.7348	0.2681	0.3217	1.0000

hEGFRvIII-CD3 bi-scFv Exposure Level	Sex	Blood urea nitrogen	Creatinine	Total Bilirubin	Sorbitol dehydrogenase	Total Protein	Albumin
0 mg/kg	F	-	-	-	-	-	-
7.5 mg/kg		0.8180	1.0000	1.0000	0.4902	0.0220	0.0955

^Hetergeneous data set with no pair-wise differences found. No p-values are given for non-parametric Dunn's test

*Hetergeneous data set with significant pair-wise differences found. No p-values are given for non-parametric Dunn's test
